# Targeting Endothelial Function to Treat Heart Failure with Preserved Ejection Fraction: The Promise of Exercise Training

**DOI:** 10.1155/2017/4865756

**Published:** 2017-06-19

**Authors:** Andreas B. Gevaert, Katrien Lemmens, Christiaan J. Vrints, Emeline M. Van Craenenbroeck

**Affiliations:** ^1^Department of Translational Pathophysiological Research and Research Group Cardiovascular Diseases, University of Antwerp, Antwerp, Belgium; ^2^Laboratory of Cellular and Molecular Cardiology and Department of Cardiology, Antwerp University Hospital (UZA), Edegem, Belgium

## Abstract

Although the burden of heart failure with preserved ejection fraction (HFpEF) is increasing, there is no therapy available that improves prognosis. Clinical trials using beta blockers and angiotensin converting enzyme inhibitors, cardiac-targeting drugs that reduce mortality in heart failure with reduced ejection fraction (HFrEF), have had disappointing results in HFpEF patients. A new “whole-systems” approach has been proposed for designing future HFpEF therapies, moving focus from the cardiomyocyte to the endothelium. Indeed, dysfunction of endothelial cells throughout the entire cardiovascular system is suggested as a central mechanism in HFpEF pathophysiology. The objective of this review is to provide an overview of current knowledge regarding endothelial dysfunction in HFpEF. We discuss the molecular and cellular mechanisms leading to endothelial dysfunction and the extent, presence, and prognostic importance of clinical endothelial dysfunction in different vascular beds. We also consider implications towards exercise training, a promising therapy targeting system-wide endothelial dysfunction in HFpEF.

## 1. Introduction

Heart failure (HF) is the most frequent cause of hospitalization in people over 65 years, and incidence is still increasing. Despite improved medical management, prognosis is grim, especially for heart failure with preserved ejection fraction (HFpEF) which has a 65% mortality rate at 5 years [1]. In contrast to heart failure with reduced ejection fraction (HFrEF), timely diagnosis of HFpEF remains a challenge and current standard therapy fails to improve prognosis [2]. Beta blockers and renin-angiotensin-aldosterone axis antagonists, drugs that mainly target the heart and have reduced mortality in HFrEF, had disappointing results in HFpEF trials [3–5]. As such, a “whole-systems” approach has been proposed, moving therapeutic focus in HFpEF away from the cardiomyocyte [6, 7].

Although HFpEF emerged as a distinct HF phenotype about three decades ago and about half of patients fall into this category, its pathogenesis remains incompletely understood. Beside advanced age, female sex, and sedentary lifestyle, HFpEF is associated with comorbidities such as arterial hypertension, diabetes, obesity, chronic obstructive pulmonary disease, and renal dysfunction [8]. Cardiac and extracardiac adjustments to these comorbidities can become maladaptive and lead to the HFpEF syndrome, with exercise intolerance as its main symptom. This maladaptation is characterized by structural changes such as myocardial hypertrophy and fibrosis, driven by a neurohormonal imbalance and systemic cytokine overexpression [9]. As a third mechanism, dysfunction of endothelial cells throughout the entire cardiovascular (CV) system has been put forward as the link between comorbidities and the pathophysiology of HFpEF. This builds on experimental evidence by Brutsaert et al. in the 1980s that the interaction between endothelial cells and cardiomyocytes directly influences diastolic function [10, 11].

Clinical endothelial dysfunction (ED) is recognized as a precursor to many CV diseases including HF [12]. Moreover, its prognostic value is proven in cohorts ranging from an unselected general population over patients at risk for CV disease (hypertension, chronic kidney disease) to patients with established CV disease [13]. Endothelial function is an independent predictor of survival in HF patients [14]. Exercise intolerance, the cardinal symptom in HFpEF, is objectively measured by peak pulmonary oxygen uptake (VO_2_peak) which is determined by the product of cardiac output and arteriovenous oxygen (O_2_) difference. Hence, both O_2_ delivery mechanisms (cardiac output, peripheral vascular function) as well as O_2_ utilizing factors (skeletal muscle) contribute to exercise intolerance [15]. Reduced endothelial-dependent vasodilation on exertion limits systemic O_2_ delivery, precipitating the switch to an anaerobic metabolism and thereby exacerbating fatigue and dyspnea [16]. ED also forms an attractive therapeutic target due to its reversibility at early stages [17]. This has shifted the search for an effective HFpEF therapy towards interventions correcting ED.

Exercise training is one of the most successful approaches to improve and even correct ED [18, 19]. Exercise-based cardiac rehabilitation programs have already earned their merit by improving symptoms and reducing mortality in various CV diseases, including HFrEF [20, 21]. The additional beneficial effects on other comorbidities and risk factors make exercise training conceptually a promising therapy for HFpEF [22].

In this review, we will focus on different aspects of ED in HFpEF. First, we briefly review the underlying molecular mechanisms leading to ED. We list the existing evidence on the presence of ED in distinct vascular beds and the clinical importance relative to HFpEF. Finally, the effects of exercise training on endothelial function are discussed, portending important implications for HFpEF treatment.

## 2. The Endothelium Is More than a Barrier

The endothelium was long considered a mere protective layer between the blood and different extravascular tissues. We now know that endothelial cells are dynamic, highly interacting cells regulating blood vessel function and homeostasis. The healthy endothelium prevents platelet and leukocyte adhesion and aggregation, inhibits smooth muscle proliferation, and regulates vascular tone through release of vasoactive substances, all of which are essential in organ perfusion [23]. Nitric oxide (NO) is the major effector molecule, formed from its precursor L-arginine by endothelial NO synthase (eNOS) in response to stimuli such as shear stress, cytokines, and platelet-derived factors. In endothelial cells, NO inhibits expression of leukocyte adhesion molecules, reducing vascular inflammation and atherosclerosis. By diffusing into platelets and vascular smooth muscle cells, NO stimulates the soluble guanylate cyclase—cyclic guanosine monophosphate—protein kinase G (sGC-cGMP-PKG) pathway, hereby inhibiting platelet aggregation and inducing vasorelaxation [23]. NO also diffuses to cardiomyocytes adjacent to coronary microvascular and endocardial endothelial cells, modulating cardiac function [24]. Finally, NO mobilizes stem cells and progenitor cells important for vascular homeostasis and repair [25].

**Figure 1:**
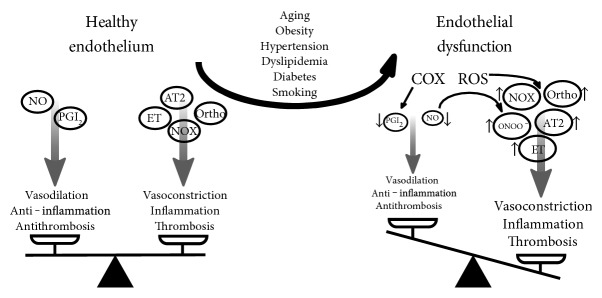
Pathophysiology of endothelial dysfunction. Healthy endothelium maintains a balance between vasodilating, anti-inflammatory, and anti-thrombotic factors on one side and vasoconstricting, inflammatory, and thrombotic factors on the other. In endothelial dysfunction, increased oxidative stress caused by comorbidities tips the balance over to a vasoconstricting, inflammatory, and thrombotic profile. AT2=angiotensin 2, COX=cyclooxygenase, ET=endothelin, NO=nitric oxide, NOX=nicotinamide adenine dinucleotide phosphate oxidase, ONOO^−^=peroxynitrite, Ortho=orthosympathetic nerve activity, PGI_2_=prostacyclin, ROS=reactive oxygen species.

In the setting of CV disease risk factors (smoking, aging, hypercholesterolemia, hypertension, hyperglycemia, and obesity), the endothelium loses these regulatory functions [26, 27]. Reactive oxygen species play an important role, reacting with NO to form toxic peroxynitrite, thereby reducing NO bioavailability. This disturbance of endothelial homeostasis can lead to a vasoconstrictory, proinflammatory, and prothrombotic phenotype at risk for CV disease [12]. The term “endothelial dysfunction” refers to these phenotypic alterations.  Figure 1 summarizes the most important molecular influences on healthy and dysfunctional endothelium.

Repair of diseased endothelium does not solely depend on proliferation of existing endothelial cells. Bone marrow-derived endothelial progenitor cells can be mobilized to sites of endothelial injury or ischemia. They are able to proliferate, exert beneficial paracrine effects through secreting vascular growth factors, and finally integrate into the endothelial layer by differentiating into endothelial cells [28, 29].

## 3. Evaluation of Endothelial Function

ED is recognized as the first—but still reversible—step to overt atherosclerosis. As such, several diagnostic evaluation methods have been developed, with the goal to identify high-risk populations and start preventive therapy early. At the other end of the spectrum, presence and severity of ED is related to a negative outcome in established coronary ischemic heart disease and HFrEF [30].

Usually, endothelial function is measured as vasodilation in response to an endothelium-specific stimulus. This includes drugs, such as acetylcholine, but a short period of local ischemia also elicits endothelium-specific hyperemia. The amount of vasodilation can be assessed invasively (e.g., coronary angiography, intravascular flow wires), although noninvasive methods are more widely used nowadays. The percentage dilation of the brachial artery in response to forearm ischemia, measured by ultrasound, is called flow-mediated dilation (FMD) [31]. The more recent EndoPAT™ device (Itamar Medical, Israel) uses a fingertip probe to measure arterial tone. The response to ischemia is calculated automatically and is called reactive hyperemia index (RHI) [32]. Details and advantages of these and other techniques to measure endothelial function have been reviewed previously [26, 33]. Generally, FMD is considered a measure of the response to shear stress in conduit vessels (macrovascular), which is largely NO dependent, while RHI measures microvascular dilatation to shear stress, which involves other vascular mediators in addition to NO [34].

## 4. Endothelial Dysfunction in HFPEF: Cause or Consequence?

Impaired coronary endothelial-dependent vasodilation was found in nonischemic dilated cardiomyopathy, highlighting the implication of the endothelium in HFrEF regardless of the presence of atherosclerosis [35]. Moreover, ED is not only limited to the coronary arteries, but is equally present in other vascular beds, indicating the systemic nature of ED in HFrEF.

In 2013, Paulus and Tschöpe hypothesized that ED plays a causal role in the development of HFpEF [10]. They postulate that the comorbid illnesses seen in HFpEF are the primary impellent of a systemic inflammatory state, leading to coronary microvascular ED. Indeed, elevated levels of inflammatory cytokines are seen in HFpEF patients [36]. In asymptomatic patients, biomarkers of inflammation predict the onset of HFpEF but not HFrEF [37]. Circulating inflammatory cytokines activate and inflame the endothelium throughout the vascular system, including the coronary microvasculature. This coronary microvascular endothelial inflammation is seen in animal models of HFpEF and in human cardiac biopsies [38, 39]. Reduced endothelium-dependent vasodilation is seen in animal models as well [40].

Reduced NO signaling from dysfunctional endothelium then influences adjacent cardiomyocytes and cardiac fibroblasts through the sGC-cGMP-PKG pathway [24]. Lower myocardial PKG content eventually leads to functional and structural cardiac changes associated with HFpEF [41]. These include delayed myocardial relaxation, increased cardiomyocyte stiffness, cardiac hypertrophy, and interstitial fibrosis [10]. Cardiac-endothelial interaction is reviewed in more detail in 6.2.

However, a two-way interaction between HFpEF and ED exists. Once HF develops, the syndrome maintains a vicious circle, further impairing endothelial function. HFpEF itself causes a systemic inflammatory state with high levels of circulating proinflammatory cytokines, increasing production of reactive oxygen species and exerting direct deleterious effects on eNOS expression [42, 43]. Neurohormonal activation in HFpEF increases oxidative stress and activates collagen synthesis [44]. Thus, HFpEF worsens system-wide ED, causing a downward spiral eventually leading to progressive HF.

## 5. Clinical Importance: Endothelial Dysfunction as Prognostic Marker in HFPEF

As there is no universally accepted cutoff for defining ED, the actual prevalence of ED in HFpEF is unknown. In community studies, endothelial function declines with age and presence of CV risk factors [45, 46]. Understandably, FMD and RHI values are lower in populations with established CV disease, including HF patients [14, 47]. In one of the first studies proving reduced RHI in HFpEF, Borlaug et al. estimated the prevalence of ED in HFpEF patients at 42% [48]. Of note, the cutoff to define ED in this study was arbitrarily chosen as RHI <2.0, which is substantially higher than the original reference value defined in coronary artery disease patients (RHI <1.67) [49]. The prevalence of ED found in the Borlaug study could as such be overestimated.

In the largest study to date, measuring endothelial function in 321 Japanese HFpEF patients, Akiyama et al. found that a RHI below the median predicted CV events [47]. For each decrease of 1.0 in RHI, CV risk increased 20%. The prognostic significance of ED in HFpEF patients was independent of clinical, echocardiographic, and neurohormonal factors. This was later confirmed in a smaller study by Matsue et al. [50]. Of note, both Japanese studies propose a prognostic cutoff value for RHI <1.63, close to the original reference value of RHI <1.67. Applied to the large Akiyama study, this implies an ED prevalence of 50% in HFpEF patients. Full details of studies measuring peripheral ED in HFpEF can be found in Table 1.

Given the central role of ED in the development of HFpEF, this estimated prevalence of ED of 42–50% seems low. However, to be more precise, 42–50% of HFpEF patients have *peripheral* ED *as defined by a given RHI cutoff*. In our opinion, the other 50% fail to show decreased RHI because of the following reasons. First, it takes time before microvascular inflammation is translated to clinically measureable disturbances in vasoreactivity. Second, the cutoff of RHI <1.63 reflects a value useful for clinical prognosis, but has not been correlated with pathophysiological changes such as endothelial inflammation and reduced NO bioavailability. Third, RHI and FMD show poor agreement which suggests different mechanisms are measured [51]. Possibly, FMD more accurately reflects reduced NO signaling, but data on FMD in HFpEF is incomplete (no large or prognostic studies), and a cutoff defining ED is not available. Perhaps, it is more correct to state that the prevalence of ED in HFpEF is hard to estimate based on current data, but almost half of patients have reduced peripheral endothelial-dependent vasodilation compared to controls, which is linked to increased CV events.

Another clinical clue to the importance of ED is the relation with exercise intolerance, objectively measured by cardiopulmonary exercise testing and determination of VO_2_peak. This is related to adverse prognosis, since VO_2_peak is one of the strongest predictors of mortality in HFpEF [52]. The Fick principle (VO_2_ = cardiac output • arteriovenous O_2_ difference) states that VO_2_peak can be limited by either a central factor, cardiac output, or peripheral O_2_ extraction. The latter is influenced by oxygenation of the blood in the lungs, O_2_ carrying capacity of the blood, appropriate distribution of blood to the peripheral tissues, and adequate tissue O_2_ extraction from the blood. A key factor is the oxygen diffusion capacity (DO_2_), which can be a limiting factor in both pulmonary and skeletal muscle O_2_ kinetics. Applying Fick's law of diffusion (VO_2_ = DO_2_ • (capillary pO_2_ – intracellular pO_2_) with pO_2_ being partial oxygen pressure) in exercising muscle, where intracellular pO_2_ is very low, the capillary pO_2_ determines the O_2_ diffusion gradient. As such, capillary pO_2_ can limit VO_2_ during exercise [53]. Adequate endothelial function is necessary for an appropriate exercise-induced increase in blood flow to the muscles [54]. As capillary pO_2_ is determined by the instantaneous balance between VO_2_ and perfusion, ED can also limit capillary pO_2_ [53]. In theory, ED can thus limit VO_2_ both by reducing capillary blood flow and limiting O_2_ diffusion.

Reduced cardiac output on exertion was long considered the main mechanism behind exercise intolerance in HFpEF [55]. Chronotropic incompetence and reduced peak stroke volume have both been implicated as the most important factor limiting VO_2_peak [56]. More recently, a peripheral limitation to exercise capacity in HFpEF has been put forward. Borlaug et al. reported reduced systemic vascular resistance and lower RHI at peak exercise in HFpEF patients compared to hypertensive and healthy controls [48]. Haykowsky et al. even suggested that a failure to increase peripheral O_2_ extraction during exercise is the predominant factor limiting VO_2_peak [57]. The rest-to-peak change in peripheral O_2_ extraction was the strongest independent predictor of VO_2_peak in their study. This was later confirmed using exercise hemodynamics and exercise echocardiography [58, 59]. Although the dominant limiting factor to VO_2_peak remains controversial, clearly peripheral elements play a role in determining exercise capacity in HFpEF. We further elaborate this finding in the next section.

## 6. Various Vascular Beds Display Endothelial Dysfunction in HFPEF

**Figure 2:**
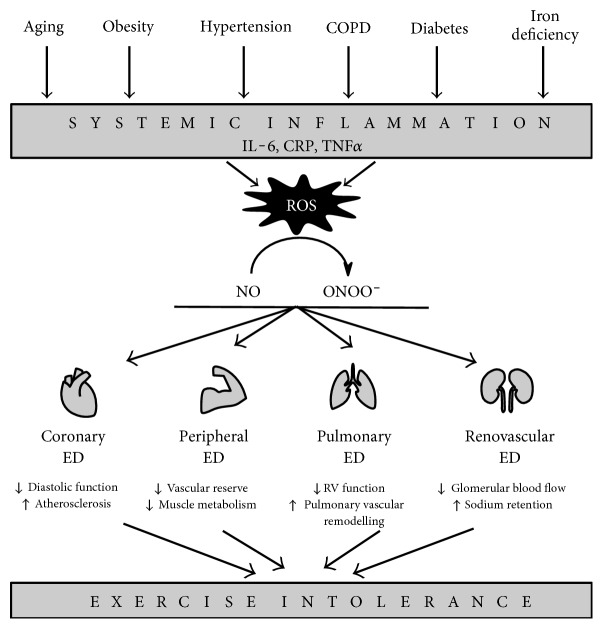
Role of system-wide endothelial dysfunction in HFpEF pathophysiology. Comorbidities induce systemic inflammation, creating oxidative stress in endothelial cells system-wide. Reduced NO bioavailability through reduction of NO to ONOO^−^ causes endothelial dysfunction. In different vascular beds, endothelial dysfunction has heterogeneous effects, which manifest as the cardinal HFpEF symptom of exercise intolerance. COPD=chronic obstructive pulmonary disease, CRP=C-reactive protein, ED=endothelial dysfunction, IL-6=interleukin-6, NO=nitric oxide, ONOO^−^=peroxynitrite, ROS=reactive oxygen species, RV=right ventricle, TNF*α*=tumor necrosis factor alpha.

Theoretically, many clinical findings related to the HFpEF syndrome could be explained by a system-wide ED, leading to alterations in several organ systems. In Figure 2, we postulate that systemic ED is the underlying pathophysiological mechanism by which HFpEF risk factors lead to exercise intolerance. Systemic inflammation induced by HFpEF risk factors creates oxidative stress at the level of the endothelium throughout the vasculature, reducing NO availability for adjacent cells pertaining to all organs implicated in exercise performance.

In what follows, we review the evidence of the presence, extent, and underlying mechanisms of ED in different vascular beds and the corresponding organs.

### 6.1. Peripheral Vasculature and Skeletal Muscle

The peripheral circulation is the preferred organ system for measuring endothelial-dependent vasodilation, because of the easy, noninvasive measurement and the good correlation with “gold standard” invasive coronary vasodilation [60]. Studies evaluating peripheral endothelial function in HFpEF are summarized in Table 1.

Evidence regarding macrovascular ED in HFpEF is conflicting. The largest study to date reported no significant difference in FMD between HFpEF patients and healthy volunteers matched for age and gender [61]. In contrast, in almost all studies assessing microvascular peripheral endothelial function through RHI measurement, HFpEF patients have evidence of microvascular ED [48, 62–65]. Also, prognostic significance for ED in HFpEF has only been proven for microvascular dysfunction [47]. Of note, many studies have different methodologies even when using the same technique for measuring endothelial function. Control groups are often heterogeneous and unmatched, few studies using FMD adhere to the most recent guidelines that state shear stimulus must be reported, [66, 67] and different cutoffs for identifying ED are used. These disparities complicate the interpretation of study results.

Besides vasodilatory dysfunction of the afferent arteries to the working muscle, reduced peripheral O_2_ extraction in HFpEF can also result from skeletal muscle dysfunction. HFpEF patients indeed have abnormalities in skeletal muscle mass, composition, capillary density, and oxidative metabolism. In contrast to the high prevalence of obesity, HFpEF patients have reduced lean leg mass [68]. This could be related to adipose tissue infiltration in muscle, which shows a similar correlation with exercise capacity. A markedly lower VO_2_peak indexed to lean body mass in HFpEF patients further confirms that abnormalities in skeletal muscle perfusion and/or metabolism contribute to exercise intolerance [69]. Mitochondria are important regulators of skeletal muscle metabolism. Recently, reductions in muscle mitochondrial content, oxidative capacity, and expression of key mitochondrial proteins were found in muscle biopsies of HFpEF patients [70]. These changes were related to VO_2_peak, emphasizing muscle mitochondrial dysfunction is likely a limiting factor to exercise capacity. Other possible underlying molecular changes could be a switch from oxidative slow-twitch type I fibers to glycolytic fast-twitch type II fibers which reduces oxidative capacity, increased muscle fatigability, and a reduction in skeletal muscle capillary density [71].

The latter is especially intriguing, as it links these skeletal muscle abnormalities to vascular dysfunction. Kitzman et al. demonstrated a severely reduced capillary-to-fiber ratio in muscles of HFpEF patients, related to VO_2_peak [72]. A lower capillary density, and hence reduced capillary blood supply, may also underlie the muscle fiber atrophy seen in animal and human HFpEF studies [69, 71]. Also, as muscle blood flow assumes an important role in limiting VO_2_ kinetics, the authors suggest a decreased O_2_ diffusion to contracting muscle limits exercise capacity in HFpEF. As mentioned above, ED could play a role in this limitation of diffusive capacity by reducing the pO_2_ driving gradient.

When leg blood flow is measured by ultrasound Doppler, HFpEF patients indeed have a reduced muscle blood flow during exercise compared to healthy controls, even at relatively low workloads of 10-15 W [73]. Stroke volume and heart rate were similar in HFpEF and control patients in this study, again implying a vascular (and not cardiac) limitation of exercise capacity. Also, HFpEF patients fail to augment peripheral O_2_ extraction during exercise with a greater increase in blood pressure than controls [59]. This suggests that a reduced vasodilatory capacity prevents appropriate distribution of blood flow during exercise, leading to limitation of exercise capacity [55]. Possibly, microvascular ED contributes more than macrovascular ED at the level of the muscle vascular bed, as Haykowsky et al. found a peripheral limitation of exercise capacity but no decrease in FMD [61, 74].

In summary, there is evidence for microvascular ED in HFpEF, predictive of long-term CV morbidity. Reports on macrovascular dysfunction are conflicting, and all studies suffer from methodological disparities. Also, HFpEF patients suffer numerous changes in skeletal muscles which correlate with reduced VO_2_peak, including mitochondrial dysfunction, fiber atrophy, and reduced oxidative capacity. Possibly, skeletal muscle abnormalities are linked to vascular dysfunction through a reduction in muscle capillary density, which limits muscle blood flow and O_2_ diffusion during exercise.

### 6.2. Heart

Traditionally, coronary endothelial function is measured by intracoronary infusion of a vasodilating substance such as acetylcholine. Subsequently, microvascular function can be estimated by measuring coronary flow reserve (CFR), the ratio of coronary blood flow after the vasodilating stimulus over blood flow at rest. In HFrEF patients, CFR correlates with VO_2_peak, invasive and echocardiographic hemodynamics, and mortality [75–77]. Tschöpe et al. measured CFR in patients with diastolic dysfunction, showing a reduced vasodilatory response to intracoronary acetylcholine infusion even before onset of HF symptoms [78]. Furthermore, invasively measured CFR is reduced in HFpEF patients and CFR correlates with echocardiographic measures of diastolic function and LV hypertrophy [76, 78, 79]. Interestingly, two studies in HFrEF patients showed no relationship between CFR and peripheral endothelial function [75, 80]. As such, different pathophysiological mechanisms may lie at the origin of coronary and peripheral ED.

As mentioned above, reduced NO bioavailability leads to both structural and functional changes in HFpEF. Structurally, HFpEF hearts are characterized by interstitial fibrosis and both macroscopic and microscopic hypertrophy [10]. Hemodynamically, diastolic dysfunction is evident as slowed ventricular relaxation on one hand and decreased compliance due to myocardial stiffness on the other hand [9].

In the normal heart, endothelial NO bursts directly modulate relaxation in a beat-to-beat way [81]. High levels of peroxynitrite (ONOO^−^), however, increase diastolic calcium content and thus delay cardiomyocyte relaxation [82]. Through its effects on sGC, NO is also able to modify cardiomyocyte stiffness and hypertrophy. sGC increases cGMP production, which in turn increases cellular PKG content. PKG acutely reduces cardiomyocyte stiffness through phosphorylation of the giant protein titin, the most important regulator of passive myocardial stiffness. Also, PKG functions as a brake on several pathways implicated in left ventricular hypertrophy. The sGC-cGMP-PKG pathway and its targets are indeed downregulated in HFpEF animals [83, 84]. Low PKG content has also been found in myocardial biopsies from HFpEF patients [41].

Finally, NO exerts direct antifibrotic effects in the heart by counteracting endothelin-1, angiotensin II, and aldosterone. Reduced NO bioavailability leaves profibrotic actions of these molecules unopposed, promoting proliferation of fibroblasts and myofibroblasts [85].

In summary, microvascular cardiac endothelium modulates diastolic function and development of LV hypertrophy and fibrosis. Coronary microvascular function, as measured by CFR, is reduced in HFpEF but does not relate to peripheral ED.

### 6.3. Lungs

Pulmonary hypertension (PHT) at rest is highly prevalent in HFpEF patients, with up to 83% affected [86]. Patients often have an exaggerated elevation of pulmonary artery pressures during exercise [87, 88]. This increased afterload on the right ventricle (RV) and the presence of common risk factors explain the high prevalence of RV dysfunction in HFpEF, which is associated with increased morbidity and mortality [89].

Passive transition of elevated end-diastolic pressure explains only part of the elevated pulmonary artery pressures in HFpEF [86]. As in patients with HFrEF and pulmonary arterial hypertension, impaired NO-dependent pulmonary vasodilation has been described in HFpEF patients. The Mayo Clinic group has spearheaded research in this field, proving abnormal RV and pulmonary artery hemodynamics both at rest and on exertion [88]. Although initially an increased pulmonary vasodilatory capacity was suggested based on dobutamine infusion [90], recent invasive measurements showed reduced exercise-induced pulmonary vasodilation in HFpEF [88].

Pulmonary arterial endothelial function was disturbed, and pulmonary artery pressures were higher in an animal infarct model of HFpEF, while aortic endothelial function and intracardiac pressures remained unaltered [91]. This could mean that pulmonary vascular ED even precedes systemic ED in HFpEF. Indeed, as the pulmonary circulation is primarily flow-driven in contrast to the pressure-driven systemic circulation, it may be more susceptible to the influence of shear stress and ED [92]. More recently, a murine model of HFpEF with PHT was established by blocking vascular endothelial growth factor receptors in obese and hypertensive rats. Oral administration of nitrite, which acts as NO donor, prevented the development of PHT but could not reverse established PHT [93]. These findings are compatible with “reversible” pulmonary ED playing an early role in the establishment of PHT, while “fixed”vascular remodeling occurs in more advanced stages.

In a cohort of 28 HFpEF patients with PHT that had severe macrovascular ED (FMD median 1.95%), Farrero et al. found a significant inverse correlation between FMD and pulmonary vascular resistance. No correlation was found with capillary wedge pressure [94]. While this does not prove a causal relationship, it is plausible that more severe HFpEF is related with more severe ED in the systemic and pulmonary vasculature, ultimately leading to PHT. This would corroborate the concept of whole-body ED in HFpEF.

PHT is also induced through reactive pulmonary vasoconstriction and vascular remodeling [95]. This process is largely mediated by NO, as pulmonary vascular reactivity is maintained by continuous local NO production [95]. A systemic reduced NO bioavailability, as found in HFpEF, causes vascular smooth muscle dysfunction in the pulmonary vasculature, paving the way for PHT [96].

Pulmonary function itself is frequently disturbed in HFpEF patients, with 59% suffering airflow limitation on spirometry [97]. As pulmonary impairment increases with symptom severity, pulmonary edema is a likely explanation. But diaphragm dysfunction may also contribute by increasing work of breathing. The diaphragm exhibits similar changes as skeletal muscle in HFpEF, including fiber atrophy, decreased oxidative capacity, impaired mitochondrial function, and increased fatigability [71]. As ED possibly underlies several skeletal muscle alterations, ED could also be a pathophysiological factor in diaphragm dysfunction, forming the link between skeletal muscle and respiratory abnormalities in HFpEF.

Pulmonary gas exchange is impaired in up to 83% of HFpEF patients, showing true O_2_ diffusion limitation at rest in 59% [97]. At exercise, diffusion abnormalities are exacerbated in HFpEF patients compared to healthy individuals [98]. These findings provide further evidence that exercise capacity is limited by O_2_ diffusion in both the systemic and the pulmonary microcirculation.

In summary, PHT is a frequent and ominous finding in HFpEF patients. Vascular remodeling and reactive pulmonary vasoconstriction, caused by a reduced systemic NO bioavailability, play an important role in its development. Spirometry, diaphragm function, and pulmonary diffusion capacity are frequently impaired in HFpEF patients. Possibly, ED plays a role by impairing O_2_ diffusion in the pulmonary microcirculation and causing adverse changes in diaphragm muscle composition similar to those in skeletal muscle.

### 6.4. Kidneys

HFpEF can induce renal dysfunction, and vice versa. Chronic kidney disease is highly prevalent in HFpEF patients (30–34% in large outcome trials) [99, 100]. Moreover, HF mortality is increased by concurrent renal impairment [101].

Clinically, endothelial function is impaired in patients with even mild chronic kidney disease, whether measured by RHI or FMD [102, 103]. Furthermore, worse endothelial function correlates with worse diastolic function on echocardiography [104]. Studies on the impact of renal disease on progression of ED in HFpEF are currently still lacking, but it is certainly an interesting field for future research [105].

HFpEF can cause renal dysfunction in different ways [106]. First, hemodynamic factors impair glomerular blood flow. Renal congestion due to elevated central venous pressure increases efferent glomerular pressure [107]. Additionally, fixed stroke volume and chronotropic incompetence reduce cardiac output on exertion, which impairs afferent blood flow [108]. The net result is decreased glomerular blood flow, leading to renovascular and glomerular injury and activating sodium retention pathways [109]. Second, the systemic inflammation that accompanies HFpEF has deleterious effects on the kidneys. Leukocyte recruitment causes renal fibrosis through transforming growth factor *ß*-mediated fibroblast stimulation. Also, systemic inflammation reduces NO bioavailability as described above. Renal blood flow is dependent on systemic NO supply, which is reduced in HFpEF [110]. In a metabolic syndrome rat model of HFpEF, degradation of peritubular and glomerular microvasculature is linked with progressive glomerulosclerosis [111]. Interestingly, in this last study, microscopic renal damage was evident *before* onset of HFpEF.

On the other hand, renal disease can also lead to HFpEF. In long-term follow-up of >8500 chronic kidney disease patients, 34% was diagnosed with new-onset HFpEF [112]. Possible mechanisms include, again, worsening endothelial function and inducing systemic inflammation [105]. Several important feedback mechanisms, regulated by the kidney and disturbed in renal failure, induce ED: vitamin D deficiency, erythropoietin deficiency, elevated parathyroid hormone levels, and phosphorus excess [113–115]. Also, the endothelium is involved in sodium handling. Sodium retention could increase intracellular sodium, which disrupts endothelial homeostasis [116]. Asymmetric dimethyl arginine, a retention product found in kidney failure, is a competitive inhibitor of eNOS and increases endothelial oxidative stress [106].

In summary, HFpEF and chronic kidney disease are mutually influencing conditions. ED is an important risk factor for both diseases, and interesting pathophysiological links exist.

## 7. Exercise Training: The Silver Lining on the Cloud

Cardiac rehabilitation programs have been a mainstay of HFrEF treatment after it was discovered that training is safe and reduces hospitalizations [21]. The evidence in HFpEF, however, is still emerging. Several medium-sized single-center studies demonstrated substantial benefit of training in HFpEF patients [117–122]. Three recent meta-analyses concluded that exercise training in HFpEF increases VO_2_peak and physical function scores [123–125]. Diastolic function (measured by E/e' ratio and left atrial volume) also improved with exercise in the landmark Ex-DHF trial [117]. These results have led to a class I, level of evidence A recommendation for exercise training in HF patients regardless of their ejection fraction in recent European Society of Cardiology HF guidelines [2]. Although no recommendations are made towards the intensity of exercise training, existing evidence suggests diverging effects of standard moderate-intensity aerobic training (at 60–70% of VO_2_peak) and high intensity interval training (adding short intervals at 80–90% VO_2_peak). In a single-center trial, high intensity training in HFrEF patients led to superior increases in VO_2_peak and ejection fraction compared to moderate training [126]. Unfortunately, these findings could not be replicated in the large multicenter SmartEx trial [127]. Of note, the majority of patients exercised below the prescribed target in the high-intensity group and above target in the moderate group. A pilot study in 15 HFpEF patients showed superior effects of high intensity interval training on exercise capacity and diastolic function [128]. However, the lack of VO_2_peak improvement in patients training at moderate intensity contrasts with the earlier studies.

The ongoing OptimEx study aims to study optimal exercise dose in 180 HFpEF patients with regard to aerobic capacity [129]. Also, this trial will reevaluate the effect of exercise training on FMD in HFpEF patients and add much-needed information on microvascular function.

In the contemporary “whole-systems” approach towards HFpEF therapy, ED forms an attractive target due to its systemic nature and reversibility in early stages. Improving ED in HFpEF can be achieved through correction of comorbidities, increasing NO bioavailability, or antioxidative therapy. Sadly, none of these approaches alone has thus far been successful in decreasing HFpEF-related morbidity or mortality. Exercise training integrates all three mechanisms, forming a promising systemically oriented therapy [7].

Both peripheral endothelial function and muscle metabolism are beneficially influenced by exercise. Exercise increases NO production by upregulating and phosphorylating eNOS through increased shear stress and vascular endothelial growth factor 2 release [19]. Exercise training also reduces oxidative stress by downregulating angiotensin receptors and nicotinamide adenine dinucleotide phosphate oxidase [130]. In addition, the anti-inflammatory and permeability decreasing properties of exercise may contribute to improvement of endothelial function [22].

Circulating progenitor cells could add to these favorable changes [131]. Endothelium-repairing endothelial progenitor cells are mobilized from the bone marrow by stimuli such as ischemia and cytokine release, under control of circulating angiogenic T lymphocytes [132, 133]. Our group has shown that the number of circulating angiogenic T lymphocytes and their functional capacity increase with exercise training, both in healthy subjects and HF patients [134]. The acute exercise-induced changes in circulating angiogenic T lymphocyte function wane with exercise training, suggesting that repetitive exercise bouts progressively lead to endothelial repair [135]. Another group has recently shown increases in endothelial progenitor cell number and function in HF patients as well [136].

Molecular determinants of exercise-induced effects specific to HFpEF are still poorly investigated. In HFpEF rats, exercise training restored endothelial-dependent vasodilation measured ex vivo in organ baths [40]. Endothelial function correlated well with eNOS expression, which was reduced in HFpEF rats and recovered after exercise training. Matrix metalloproteinase activity, which is an indirect measure of extracellular matrix degradation and thus vessel wall modulation, was increased in HFpEF and blunted by exercise training while the endothelial cell layer remained intact. This suggests exercise-induced vascular changes extend beyond the endothelium.

In a secondary analysis of the Ex-DHF trial, circulating cytokines and hormones were analyzed in HFpEF patients before and after training [137]. Inflammatory cytokines (interleukins 1*ß*, 6, and 10 and tumor necrosis factor alpha) showed no change with exercise. Interestingly, levels of the growth hormone releasing peptide ghrelin, which inhibits cardiomyocyte and endothelial cell apoptosis in vitro, increased by exercise training. Clearly, molecular determinants underlying the exercise-induced benefits in HFpEF deserve further in-depth exploration.

Clinically, peripheral endothelial function shows improvement after exercise training in patients with CV risk factors, coronary atherosclerosis, and HFrEF [138–140]. Of note, when comparing high intensity interval training to moderate training in HFrEF, endothelial function (as measured by FMD) and mitochondrial function (determined from muscle biopsies) improved only by high intensity training [126]. In HFpEF patients, Haykowsky et al. found that exercise training can increase peripheral O_2_ extraction. The increase in VO_2_peak was almost entirely attributable to an improvement in peripheral function (i.e., improved vascular and/or skeletal muscle functions) [141]. In a study by Fu et al., aerobic interval training increased muscle perfusion and muscle O_2_ extraction in HFpEF patients. This increase in muscle vascular function was the only significant predictor of VO_2_peak. Interestingly, this phenomenon was not seen in HFrEF patients, for whom improved cardiac output was the only predictor of VO_2_peak [142].

Conversely, Kitzman et al. could not demonstrate an improvement of FMD after training HFpEF patients, despite an increase in VO_2_peak [74]. A possible confounder could be that FMD was measured in the postprandial state in the Kitzman study, while guidelines advise to assess FMD in a fasting state because of a significant influence of food ingestion [67, 143]. In addition, the intensity of the exercise training protocol in this study was rather moderate and therefore could have failed to induce changes in macrovascular endothelial function.

There is no data regarding the effects of training on coronary, pulmonary, or renal ED in HFpEF patients. However, studies in patients with other CV diseases suggest exercise training is indeed able to improve regional endothelial function. Coronary endothelial function is improved by cardiac rehabilitation in dilated cardiomyopathy and coronary atherosclerosis [138, 144]. In patients with chronic kidney disease, changes in several molecular markers (asymmetric dimethyl arginine, glutathione, and lipid peroxidation products) suggest increased NO bioavailability through exercise training [106]. Unfortunately, Van Craenenbroeck et al. found that exercise training did not improve FMD nor cellular markers of vascular function, despite an increase in VO_2_peak [102]. However, data on microvascular function is lacking.

## 8. Future Directions

Considering HFpEF as a multisystem syndrome rather than an isolated cardiac disease could lead us to alternative research approaches and eventually to successful therapies. The heterogeneity of the HFpEF patient population has frequently been cited as one of the reasons major trials have failed to prove a benefit for pharmacological treatment [145]. Efforts to subdivide HFpEF patients into different phenotypes have only started recently [146–148]. In the spectrum of HFpEF as a multisystem pathology, some patients seem younger and suffer less cardiac impairment, some have important metabolic disorders and more severe cardiac disease including RV and pulmonary vascular involvement, and others have a predominant renal dysfunction. Importantly, prognosis between phenotypes differs substantially [147]. The greatest challenges for future HFpEF research will be to correctly stratify patients into phenogroups and to design clinical trials accordingly. Whether endothelial function measurement could aid in identifying the correct HFpEF phenotype in patients is still unknown.

Also, a one-size-fits-all therapeutic approach is probably not the best strategy for the heterogeneous HFpEF population. A treatment algorithm based on presence of different comorbidities has recently been proposed [146]. Keeping in mind the important effects of even low-level exercise, matching or stratifying groups for physical activity seems reasonable when designing HFpEF trials, although maintaining statistical power will require a delicate balance. Rather, we support further subdividing of HFpEF based on large phenotyping studies to better characterize this heterogeneous population. Clinical trials could then be focused on a well-defined subgroup, eliminating confounding by other phenotypes.

**Figure 3:**
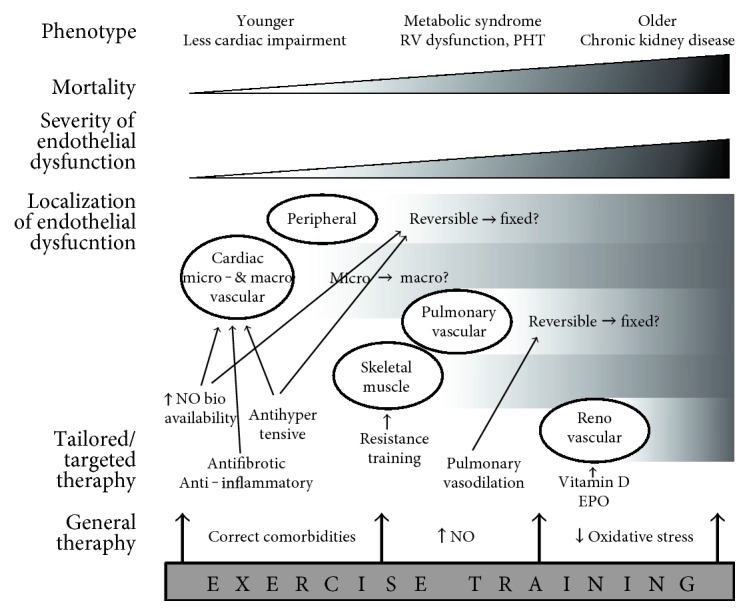
Possibilities for exercise training and targeted therapies depending on HFpEF phenotype. Cardiac ED is an early hallmark in all HFpEF patients. In older patients, pulmonary and renal vasculature are more frequently involved, and mortality is higher. HFpEF therapy could be tailored for each phenotype. Younger patients could still benefit from correction of comorbidities, preventing further systemic inflammation and ED. Increasing NO bioavailability, antifibrotic, or anti-inflammatory therapy could also be useful in early stages. Pulmonary vasodilation can only be effective when pulmonary vascular ED is manifested and still reversible. Exercise training has possible benefits at each stage, as it is able to correct comorbidities (weight loss, better glycemic control), increase NO bioavailability, and reduce systemic oxidative stress. EPO=erythropoietin, NO=nitric oxide, PHT=pulmonary hypertension, RV=right ventricle.

Unravelling the beneficial effects of exercise training in HFpEF could lead to patient-specific new therapies. Such a tailored approach can be useful in patients who are unable to exercise, or as add-on to a training program. Pharmacological or nonpharmacological correction of comorbidities, increase of NO bioavailability, and antioxidative therapy are possible targets, some of which are being explored in clinical trials already [119, 149]. These can be combined with exercise training to compose a truly personalized treatment for each patient (Figure 3).

## 9. Conclusions

HFpEF is a multisystem pathology. Cardiac dysfunction is not the sole causative factor, but interacts with a heterogeneous range of organ dysfunctions, including pulmonary, renal, peripheral vascular, and skeletal muscle dysfunctions. Endothelial dysfunction could be a central mechanism in this system-wide CV maladaptation, as such it forms an attractive target for future HFpEF therapies. Exercise training is thus far the only therapy with proven beneficial effects in HFpEF. While exercise training does not improve macrovascular ED in HFpEF, evidence does suggest peripheral vascular and/or skeletal muscle function is enhanced. This warrants a shift in both fundamental and clinical research towards endothelial-targeted therapies, including exercise training, in the search for an effective therapeutic strategy for HFpEF.

## Figures and Tables

**Table 1:** Studies assessing peripheral endothelial function in HFpEF patients compared to a control population.

Reference	Technique	Outcome variable	Study design	Number of patients	Number of HFpEF patients	Control groups	Result	*P* value
*Studies assessing macrovascular endothelial function*								
Hundley et al., 2006 [150]	FMD (magnetic resonance)	% change in femoral artery area	Case-control	30	9	Healthy, matched for age	FMD comparable in HFpEF and healthy,(12 ± 1 versus 14 ± 2%), no difference in shear rate	ns
Haykowsky et al., 2013 [61]	FMD (ultrasound)	% dilatation brachial artery	Case-control	111	60	Young healthy group, matched for gender Old healthy group, matched for age and gender	FMD better in young healthy (6.13 ± 0.53%) FMD comparable in HFpEF and old healthy (3.64 ± 0.28 versus 4.00 ± 0.38%),	<0.001 versus young ns versus old
Farrero et al., 2014 [94]	FMD (ultrasound)	% dilatation brachial artery	Case-control	70	28	Hypertensive, matched for age	FMD significantly lower in HFpEF + PHT (1.95 [−0.81–4.92] versus 5.02 [3.90–10.12] %), no difference in shear rate (*p* = 0.47)	0.002
Kishimoto et al., 2017 [151]	FMD (ultrasound)	% dilatation brachial artery	Case-control	206	41	Subjects without heart failure, unmatched	FMD significantly lower in HFpEF (2.9 ± 2.1 versus 4.6 ± 2.7%)	0.0002
*Studies assessing microvascular endothelial function*								
Balmain et al., 2007 [65]	Laser Doppler; venous occlusion plethysmography	Perfusion units; mL/100mL blood	Case-control	36	12	Coronary heart disease patients, unmatched	Cutaneous blood flow lower in HFpEF patients, venous capacitance was not different versus control∗	<0.001
Borlaug et al., 2010 [48]	RHI (PAT)	Ln (PAT ratio 60–120 sec)	Case-control	50	21	Hypertensive group; Healthy group; both matched for age and gender	RHI significantly lower in HFpEF versus healthy (0.85 ± 0.42 versus 1.33 ± 0.34), but not in HFpEF versus hypertensive (0.85 ± 0.42 versus 0.92 ± 0.38)	<0.05∗ ns
Akiyama et al., 2012 [47]	RHI (PAT)	Ln (PAT ratio 90–150 sec)	Prospective cohort	494	321	Healthy, matched for age, gender, and presence of hypertension and diabetes mellitus	RHI significantly lower in HFpEF (0.53 ± 0.20 versus 0.64 ± 0.20)	<0.001
Vitiello et al., 2014 [152]	Venous occlusion plethysmography	mL/100mL blood	Case-control	32	18	Healthy, unmatched	Venous capacitance was not different versus healthy	ns
Yamamoto et al., 2015 [64]	RHI (PAT)	Not reported	Case-control	128	64	Healthy, matched for age, gender and comorbidities	RHI significantly lower in HFpEF (1.70 [1.55 – 1.88] versus 2.01 [1.64 – 2.42])	<0.001
*Studies assessing both macro- and microvascular endothelial function*								
Maréchaux et al., 2016 [62]	FMD (ultrasound)	% dilatation brachial artery	Case-control	90	45	Hypertensive, matched for age, sex, and presence of diabetes mellitus	FMD significantly lower in HFpEF patients (3.6 [0.4 – 7.4] versus 7.2 [3.2 – 12.7] %)	0.001
	RHI (Laser Doppler)	Perfusion units					Cutaneous blood flow lower in HFpEF patients (135 [104 – 206] versus 177 [139 – 216] units)	0.03
Lee et al., 2016 [63]	FMD (ultrasound)	% dilatation brachial artery	Case-control	48	24	Healthy, matched for age, sex, and brachial artery diameter	FMD lower in HFpEF (3.06 ± 0.68 versus 5.06 ± 0.53), but no difference in FMD when corrected for shear rate	ns
	RHI (ultrasound)	Blood flow AUC after cuff release					AUC lower in HFpEF (454 ± 35 versus 659 ± 63 mL/min)	0.03

AUC: area under the curve; FMD: flow mediated dilatation; HFpEF: heart failure with preserved ejection fraction; Ln: natural logarithm; ns: not significant; PAT: peripheral arterial tonometry; PHT: pulmonary hypertension; RHI: reactive hyperemia index; ^∗^exact numbers not reported.
